# Early oral colonization of streptococcus mutans and lactobacilli species in infants: Investigating the relationship with maternal and environmental factors, from birth through the sixth month

**DOI:** 10.12669/pjms.40.9.9602

**Published:** 2024-10

**Authors:** Cansu Ay, Huseyin Karayılmaz, Ayse Cengiz

**Affiliations:** 1 Cansu Ay, DDS, PhD, Pediatric Dentist, Private Practice, Yalova, Turkiye; 2 Huseyin Karayılmaz, DDS, PhD, Professor, Akdeniz University, Faculty of Dentistry, Department of Pediatric Dentistry, Antalya, Turkiye; 3 Ayse Cengiz, DDS, Research Assistant, Akdeniz University, Faculty of Dentistry, Department of Pediatric Dentistry, Antalya, Turkiye

**Keywords:** Infant, *Lactobacillus*, Newborn, *Streptococcus Mutans*, qRT-PCR

## Abstract

**Objective:**

To assess the presence of *Streptococcus Mutans* (*S.mutans*) and *Lactobacillus* species (*LB*) of newborn-mother pairs using the real time polymerase chain reaction (qRT-PCR).

**Method:**

Subjects were selected from the patients followed in the Neonatology Clinic of Akdeniz University’s Faculty of Medicine between the years 2017-2018. First samples collected within 48 hours after birth, and second samples were at six months. The samples were analyzed for the presence of *S.mutans* and *LB* using qRT-PCR. Mothers’ smoking habits, education level, occupation, oral hygiene habits, DMFT scores and dietary history; Babies’ delivery type, birth weight, feeding type, oral hygiene practices, feeding habits, bottle usage, pacifier usage, consumption of sugary foods, were also recorded. The effect of factors related to both mothers and infants was examined comparatively.

**Results:**

*S.mutans* DNA was detectable in 87% and *LB* DNA was detected in 37% mothers, while it was undetected in 63% mothers at the first sampling. *S.mutans* was detected in 37% and, while *LB* was detected in 5% of the newborns in the first 48 hours of their life. At the second sampling, the *S.mutans* and *LB* levels in infants have increased, while there has been no significant change in mothers. A significant relationship was found only between the increase in *S.mutans* in infants and the presence of erupted teeth.

**Conclusion:**

*S.mutans* and *LB* were able to colonize on the oral mucosal surfaces of edentulous newborns, with the counts of both bacteria increasing significantly with tooth eruption.

## INTRODUCTION

*Streptococcus mutans* (*S.mutans*) and *Lactobacillus* species (*LB*) are the bacteria responsible for the onset and progression of caries.^1^,^2^ The mother, who is often the primary caregiver, is typically the primary source of cariogenic bacteria in infants.^3^ While it was previously believed that these bacteria are not present in newborns until their teeth begin to erupt, advancements in the fields of microbiology and genetic science have revealed the presence of *S.mutans* in infants as young as three months old.^4^

Many studies have examined the transmission and colonization of cariogenic bacteria and the composition of children’s oral flora in relation to various factors.^3^-^5^ The primary techniques used to detect the presence of oral microorganisms for this purpose are bacterial culture and deoxyribonucleic acid (DNA) hybridization.^6^ However, the bacterial culture system has a significant drawback in terms of its inability to reliably detect specific microorganisms in samples collected in small quantities. Thus, the use of real-time quantitative polymerase chain reaction (qRT-PCR) has emerged as a more rapid and sensitive method for both detecting and quantifying oral bacteria via a single assay.^6^-^8^

The present study sought to assess the oral microflora of newborns and their mothers with regard to the presence of *S.mutans* and *LB* using the qRT-PCR technique as well as to investigate the interactions with each other.

The H_0_ hypothesis is that *S. mutans* and *LB* can colonize in edentulous newborns. The H_1_ hypothesis is that the *S*. *mutans* and *LB* counts of the newborns are related to maternal sociodemographic factors**.**

## METHODS

Subjects were selected from the patients followed in the Neonatology Clinic of Akdeniz University’s Faculty of Medicine between the years 2017-2018. The study included 60 newborn-mother pairs. Mothers were informed, and they each signed a consent form. Medical histories and sociodemographic information of both the mother and the baby were recorded. Mothers with a history of systemic disease or drug usage, as well as newborns suspected of having systemic disease or syndromes, were excluded from the study.

### Ethics statement:

This study is ethically approved by the Akdeniz University Clinical Research and Ethics Committee (Ref. No.: 70904504/400, Date: November 06, 2017) and conforms to the recognized required standards of the Declaration of Helsinki.

Saliva samples were collected from 60 newborn-mother pairs by a single operator (CA) using liquid base swabs (eSwab, Copan, Italy). Swabs were gently rubbed along the buccal mucosa, gingiva, hard and soft palate, tongue surface, entire lingual surface of the mandibular crest, and retromolar area for approximately 60 seconds. The swabs then were placed into sealed tubes and stored upright at -20°C until taken to the laboratory. Samples were transported to the laboratory in a thermal isolation bag.

A secondary sample collection was performed at the sixth month. The second saliva samples were collected using the same procedure and stored and transported under the same conditions as the first samples. All the laboratory procedures were conducted in a molecular genetics research and development laboratory setting (Krosgen Biotechnology Research and Development Ltd. Co., Istanbul, Turkey).

### Isolation of DNA fragments from the samples:

A bacterial DNA isolation kit (AMBRD Laboratories, Istanbul) with 50 reactions was employed. The final volume of DNA isolates was set at 100 μl. For the intermediate steps, the protocol specified by the kit’s manufacturer was strictly followed, resulting in the completion of the DNA isolation in approximately 30-45 minutes.

### qRT-PCR process for the DNA isolates:

The LightCycler Nano Real-Time PCR System (Roche Diagnostics GmbH, Mannheim, Germany) and clear LightCycler Tube Strips (Roche Diagnostics GmbH, Mannheim, Germany), which were supplied in sets of eight tubes, were used. To detect the presence and quantify the numbers of *S.mutans* and *LB* in the samples, primers were employed for the purpose of amplification. The primer design was based on sequences obtained from the National Center for Biotechnology Information (NCBI).^9^ The primers used and the regions of the genes to be amplified were as follows:

For S.mutans, to amplify a 168 bp region in the Glucosyltransferase-I gene:

- “Forward” 5’ TGGTGCTCAATCAATTAACGGT 3’
- #x201C;Reverse” 5’ AGTGGTGTATGGCGTCACTT 3’
  For *LB* species, to amplify a 195 bp region in the 16S ribosomal RNA gene:
- “Forward” 5’ TCGCATCGCATAACCGCT 3’
- “Reverse” 5’ ATTCACTTGCAGGCAATACACT 3’

In accordance with the manufacturer’s instructions, the reactions were carried out via a three-cycle method. The contents of the tubes prepared for the qRT-PCR were as follows: SYBR Green Master Mix 2X: 10 μl; Primer Forward: 1 μl, 500 M; Primer Reverse: 1μl, 500 M; Water 3μl; Sample 5μl. To control for any potential contamination, a negative control was included. To accurately determine the bacteria count, a standard curve was established before proceeding with the PCR. This curve was used for the calculations.

Following the standard curve study, qRT-PCR was performed on each sample, with numerical data obtained based on the Ct values. The process was transferred to the computer as an image, and the results were obtained via numerical calculations using the LightCycler Nano Software program. The results were recorded as the DNA copy counts per milliliter (copies/mL). The samples were amplified according to the instructions of the kit used in the qRT-PCR device. All the stages of the reaction, temperature degrees, and durations are shown in Table-I.

**Table-I:** qRT-PCR steps.

Temperature (°C)	Second	Cycle	Process
95	120	1 Cycle	Polymerase activation
95	5	40 Cycle	Denaturation
60	10		Annealing
72	15		Extension

### Statistical Analyses:

The obtained data were entered into the SPSS package program (SPSS 18.00 for Windows, Chicago, IL, USA), descriptive statistics (minimum, maximum, mean, standard deviation, etc.), and comparison tests were carried out.

In the comparison of quantitative (quantitative) data, the “Student T” test was used when parametric conditions were met (Levene’s Test), one-way analysis of variance was used in repeated measurements of groups, and the Tukey HSD multiple comparison test was used in subgroup comparisons. In cases where parametric conditions could not be met, Kruskal-Wallis, Mann-Whitney U, Wilcoxon, and chi-square (χ2) tests were used to analyze qualitative data and compare groups. Survival time analysis was performed using the Kaplan-Meier Test. The results were evaluated at the 95% confidence interval, at the p<0.05 significance level.

### Consent:

All the mothers were informed about the purpose and procedures of the study, and they each signed a consent form.

## RESULTS

### Socio-demographic and medical characteristics at the first sampling:

Among 60 mothers 70% were housewives, while the remainder were active employees. The mean maternal age was 27.5 years (range: 18-40 years). Education status was high education for 17% of the mothers, medium for 37%, and low for 46%. Smoking during pregnancy observed in 13% of the mothers, none of them reported consuming alcohol. The mean DMFT score for mothers was 5.45±3.8. Regarding oral hygiene, 33.3% of the mothers brushed their teeth twice a day, 30% brushed once a day, and 36.7% did not engage in regular tooth brushing habits.

Thirty-six babies were male and 24 were female. Vaginal delivery rate was 47% whereas 53% were delivered via C-section. Preterm babies comprised 17%. Low birth weight was observed in 5% of the babies. Samples were collected from 15% of the newborns within the first six hours after delivery, from 63% between six and 24 hours after delivery, and from 22% between 24 and 48 hours after delivery.

### Bacterial acquisition at the first sampling:

*S.mutans* DNA was detectable in 87% of the mothers with a mean of 0.89×106±3.12×106 copies/mL. When the *S.mutans* DNA copies of the mothers were compared by age, a statistically significant difference was found (p=0.017), which was attributed to the higher *S.mutans* DNA counts among the mothers in the 18–20 age group. *LB* DNA was detected in 37% of the mothers, with a mean of 0.16×104±1.10×104 copies/mL. When the *LB* DNA copies were compared among the mothers’ age groups, no statistically significant difference was observed. The *LB* DNA copies in the smoking mothers were significantly higher when compared with the non-smoking mothers (p=0.008).

*S.mutans* was detected in 37% of the 60 newborns, while *LB* was detected in only 5% of the newborns at the first 48 hours of their life, thus the H_0_ hypothesis is accepted. The mean *S.mutans* DNA count was 0.40×10^3^±1.17×10^3^ copy/mL, while the mean *LB* DNA count was 7.21±49.80 copy/mL. There was no statistically significant difference between the infants delivered vaginally and C-section. The effects of the maternal and infantil sociodemographic-medical characteristics on the infants’ *S.mutans* and *LB* counts were not statistically significant (Table-II and Table-III). While there was a statistically significant difference (p=0.03) between the *S.mutans* counts obtained from the mothers and infants, there was no significant difference between the *LB* counts (p=0.247).

**Table-II:** Distribution of Infant’s Mean S. mutans and LB counts based on baby-related factors at the first and second sampling.

			First Sampling		Second Sampling	

Groups		N	Baby SM	Baby LB	Baby SM	Baby LB

			(Mean±SD)×10^x^copy/mL	Mean±SD kopya/mL	(Mean±SD)×10^x^copy/mL	(Mean±SD)×10^x^copy/mL
Gender	Male	24	(0,25±0,38)×10^3^	16,70±78,51	(3,58±7,14)×10^3^	(1,09±2,49)×10^2^
	Female	16	(0,64±2,03)×10^3^	2,00±8,00	(1,66±3,77)×10^3^	(0,65±2,12)×10^2^
P			0,365	0,462	0,332	0,561
Delivery Type	Normal	18	(0,67±1,90)×10^3^	23,16±90,61	(3,71±8,32)×10^3^	(0,32±1,37)×10^2^
	C-Section	22	(0,19±0,37)×10^3^	0,72±3,41	(2,07±3,23)×10^3^	(1,40±2,82)×10^2^
P			0,253	0,252	0,401	0,146
Birth Weight	LBW	4	(0,19±0,39)×10^3^	0±0	(0,16±0,33)×10^3^	(1,45±2,90)×10^2^
	NBW	36	(0,43±1,37)×10^3^	12,02±64,20	(0,29±0,61)×10^3^	(0,72±1,96)×10^2^
P			0,741	0,713	0,677	0,505
Delivery Time	Preterm	10	(0,30±0,49)×10^3^	4,80±10,79	(1,55±3,01)×10^3^	(0,58±1,83)×10^2^
	Term	30	(0,44±1,49)×10^3^	12,83±70,29	(3,23±6,74)×10^3^	(1,03±2,49)×10^2^
P			0,776	0,723	0,454	0,601
Birth order in the family	1st	20	(0,58±1,81)×10^3^	21,65±85,87	(2,47±4,09)×10^3^	(1,29±2,94)×10^2^
	2nd	13	(0,25±0,48)×10^3^	0±0	(4,20±9,16)×10^3^	(0,84±1,79)×10^2^
	3rd	4	(0,08±0,16)×10^3^	0±0	(1,94±3,89)×10^3^	0±0
	4th	3	(0,33±0,34)×10^3^	0±0	(0,21±0,38)×10^3^	0±0
P			0,862	0,753	0,725	0,672
Sampling Time	Within 6 hours after birth	6	(1,52±3,23)×10^3^	5,33±13,06	(2,45±5,25)×10^3^	(1,57±3,86)×10^2^
	Between 6 to 24 hours after birth	24	(0,16±0,37)×10^3^	16,70±78,51	(3,25±7,08)×10^3^	(0,66±1,70)×10^2^
	Between 24 to 48 hours after birth	10	(0,31±0,52)×10^3^	0±0	(1,98±3,65)×10^3^	(1,13±2,70)×10^2^
P			0,071	0,755	0,852	0,664

SM: S. mutans; LB; Lactobacillus spp; LBW: Low Birth Weight; NBW: Normal Birth Weight.

**Table-III:** Distribution of S. mutans and LB counts of mother-newborn pairs, based on maternal factors at the first and second sampling.

			First Sampling					Second Sampling			

			Mother SM	Mother LB	Newborn SM	Newborn LB	N	Mother SM	Mother LB	Infant SM	Infant LB

	Groups	N	(Mean±SD) ×10^6^Copy/mL	(Mean±SD) ×10^3^Copy/mL	(Mean±SD) ×10^3^Copy/mL	Mean±SD Copy/mL		((Mean±SD) ×10^6^Copy/mL	(Mean±SD) ×10^3^Copy/mL	((Mean±SD) ×10^3^Copy/mL	(Mean±SD) ×10^2^Copy/mL
Age Groups	18-20 Years old	5	(5.16±9.14)×10^6^	(0.75±1.02)×10^3^	(0.06±0.14)×10^3^	0.00±0.00	4	(0.17±0.33)×10^6^	(0.40±0.64)×10^3^	(1.80±3.25)×10^3^	(2.92±4.48)×10^2^
	21-25 Years old	21	(1.10±2.52) ×10^6^	(0.32±0.80) ×10^3^	(0.61±1.78) ×10^3^	2.28±7.64	14	(1.42±3.01)×10^6^	(0.57±0.83)×10^3^	(2.61±4.55)×10^3^	(0.14±0.55)×10^2^
	26-30 Years old	15	(0.12±0.28)×10^6^	(5.91±22.05)×10^3^	(0.30±0.58)×10^3^	0.00±0.00	9	(0.19±0.40)×10^6^	(4.58±13.35)×10^3^	(2.71±3.68)×10^3^	(0.64±1.93)×10^2^
	31-35 Years old	14	(0.21±0.27)×10^6^	(0.07±0.22)×10^3^	(0.15±0.24)×10^3^	27.50 ±102.89	10	(0.11±0.17)×10^6^	(0.45±0.82)×10^3^	(4.36±10.3)×10^3^	(0.88±1.98)×10^2^
	36-40 Years old	5	(0.017±0.02)×10^6^	(0.003±0.007)×10^3^	(0.84±1.48)×10^3^	0.00±0.00	3	(0.09±0.15)×10^6^	(0.10±0.18)×10^3^	(0.21±0.38)×10^3^	(2.78±4.82)×10^2^
	P		0.017	0.577	0.656	0.562		0.378	0.585	0.868	0.159
Education	Primary	28	(0.65±1.05)×10^6^	(3.31±16.14)×10^3^	(0.33±0.74)×10^3^	0.57±3.02	16	(1.85±2.83)×10^6^	(2.90±9.95)×10^3^	(3.20±8.39)×10^3^	(0.49±1.51)×10^2^
	High	22	(1.59±5.00)×10^6^	(0.03±0.06) ×10^3^	(0.61±1.74)×10^3^	0.00±0.00	16	(0.23±0.57)×10^6^	(0.35±0.69)×10^3^	(2.71±4.01)×10^3^	(1.44±3.05)×10^2^
	University	10	(0.06±0.01)×10^6^	(0.008±0.01)×10^3^	(0.12±0.27)×10^3^	41.70 ±121.04	8	(0.14±0.20)×10^6^	(0.45±0.89)×10^3^	(2.23±4.12)×10^3^	(0.72±2.05)×10^2^
	P		0.381	0.569	0.513	0.054		0.267	0.478	0.933	0.512
Occupation	Working	18	(0.31±0.51)×10^6^	(0.19±0.50)×10^3^	(0.10±0.20)×10^3^	23.16±90.61	12	(0.17±0.36)×10^6^	(0.28±0.75)×10^3^	(0.96±1.82)×10^3^	(0.94±2.49)×10^2^
	Housewife	42	(1.14±3.70)×10^6^	(2.30±13.18)×10^3^	(0.52±1.38)×10^3^	0.38±2.46	28	(0.77±2.20)×10^6^	(1.87±7.53)×10^3^	(3.60±7.01)×10^3^	(0.90±2.30)×10^2^
	P		0.348	0.503	0.210	0.105		0.361	0.475	0.208	0.961
Smoking	Yes	8	(0.86±0.95)×10^6^	(11.08±30.1)×10^3^	(0.15±0.28)×10^3^	0.00±0.00	5	(2.47±4.85)×10^6^	(0.75±0.97)×10^3^	(0.56±0.95)×10^3^	(0.41±0.93)×10^2^
	No	52	(0.90±3.33)×10^6^	(0.22±0.62) ×10^3^	(0.43±1.25)×10^3^	8.32±53.48	35	(0.32±0.78)×10^6^	(1.48±6.76) ×10^3^	(3.13±6.39) ×10^3^	(0.99±2.46)×10^2^
	P		0.974	0.008	0.527	0.664		0.014	0.811	0.381	0.613
O. Hygiene	Twice a day	20	(1.41±4.75)×10^6^	(0.14±0.45)×10^3^	(0.67±1.93)×10^3^	19.25 ±86.08	16	(1.21±2.83) ×10^6^	(0.62±0.79) ×10^3^	(4.49±8.50) ×10^3^	(1.20±2.50) ×10^2^
	Once a day	18	(0.14±0.23)×10^6^	(5.09±20.11)×10^3^	(0.33±0.53)×10^3^	1.77±7.54	11	(0.11±0.12)×10^6^	(0.23±0.52)×10^3^	(1.28±3.94)×10^3^	(0.52±1.74)×10^2^
	≤ 3 times a week	22	(1.05±2.47)×10^6^	(0.26±0.59) ×10^3^	(0.20±0.36)×10^3^	0.72±3.41	13	(0.24±0.58)×10^6^	(3.32±11.09)×10^3^	(2.03±2.93) ×10^3^	(0.89±2.64)×10^2^
	P		0.447	0.295	0.421	0.423		0.230	0.414	0.349	0.767
DMFT	<1	6	(0.65±1.58)×10^6^	0.00±0.00	(0.10±0.21)×10^3^	64.16 ±157.17	5	(0.20±0.42)×10^6^	(0.57±0.73)×10^3^	(4.22±4.03)×10^3^	0±0
	1-3	16	(0.08±0.13)×10^6^	(0.28±0.70) ×10^3^	(0.54±2.02)×10^3^	0.00±0.00	10	(0.22±0.43)×10^6^	(0.31±0.83)×10^3^	(1.86±4.33)×10^3^	(1.05±2.65)×10^2^
	4-6	18	(1.02±2.65)×10^6^	(4.96±20.13)×10^3^	(0.28±0.54)×10^3^	1.77±7.54	12	(1.13±3.18)×10^6^	(0.43±0.77)×10^3^	(2.29±3.58)×10^3^	(0.90±1.83)×10^2^
	7-10	12	(2.15±6.09)×10^6^	(0.28±0.84)×10^3^	(0.50±1.02)×10^3^	1.33±4.61	7	(0.71±1.50)×10^6^	(0.40±0.52)×10^3^	(5.03±12.4)×10^3^	(2.18±3.88)×10^2^
	11	8	(0.53±0.58)×10^6^	(0.39±0.70)×10^3^	(0.44±0.55)×10^3^	0.00±0.00	6	(0.33±0.50)×10^6^	(6.97±16.27)×10^3^	(1.66±3.12)×10^3^	0±0
	P		0.537	0.696	0.931	0.062		0.798	0.243	0.797	0.447

SM: S. mutans; LB; Lactobacillus spp; O.Hygiene: Oral Hygiene practices; DMFT: decayed, missing, filling tooth index.

### Socio-demographic and medical characteristics at the second sampling:

Due to the exclusion of 20 subject pairs for various reasons, the second sampling was performed on 40 subject pairs. No changes were observed in the mothers. The changes in the oral hygiene frequencies of the 40 mothers were also not statistically affected. Sixteen infants had erupted teeth. The average weight of the infants was 7.79±0.92 kg. Oral hygiene care for the infants was “every day” for seven infants, “three times a week or less” for 17 and “never” for 16 of them. Frequency of bathing for infants was as follows: 27.5% twice a week, 62.5% once a week, 10% every 10–15 days. Thirty-four infants were cared by their own mother, while six were cared by a caregiver or another family member. Seventeen infants were breastfed, while 23 infants were breastfed and given complementary foods.

Bottle usage was observed in 17 infants. Pacifier usage was observed in 11 infants and it was found that the pacifier was either being dipped in sugary food or placed in mouth by their caregivers before giving the baby. Tasting the spoon used to feed the infants before feeding them and kissing the baby’s lips was categorized under the same heading and those habits was present in 37.5% of mothers/caregivers. Sugary food consumption was detected in 70% of the infants.

### Bacterial acquisition at the second sampling:

*S.mutans* was detectable in 95% of 40 mothers. The mean *S.mutans* DNA count was 0.59±1.86×106 copies/mL. *LB* DNA was found in 35%. The mean *LB* DNA count was 1.29±6.34×103 copies/mL. *S.mutans* was detected in 45% of 40 infants and *LB* was detected in 17% of them. The average *S.mutans* DNA count was 2.81±6.04×10^3^ copy/mL, while the average *LB* DNA count was 0.92±2.33×10^2^ copy/mL.

The *S.mutans* and *LB* counts of mothers were not affected by mothers’ age groups, educational levels, employment status and oral hygiene frequencies. However, the smoking mothers had a significantly higher *S.mutans* count then the non-smoking mothers(p=0.014) (Table-III). *S.mutans* DNA counts of the mothers and infants were significantly different(p=0.049), although *LB* DNA counts were not (p=0.237). The mothers’ age, education level, occupation, tooth brushing frequency, and DMFT scores did not have a statistically significant effect on the infants’ *S.mutans* and *LB* counts (Table-III). Thus the H_1_ hypothesis is rejected.

### Comparison of the first and second sampling data:

An insignificant decrease in the *S.mutans* counts and an insignificant increase in the LB counts was observed in mothers. For the infants, there was a statistically significant increase in both the *S.mutans* and *LB* counts (Table-IV). To identify the cause of this increase, we examined the effects of the mother’ and infants’ characteristics. The infants with erupted teeth had a significantly higher number of *S.mutans* bacteria(p=0.005) (Table-V).

**Table-IV:** Comparison of the first and second sampling mean S.mutans and LB levels.

Sampling	S. mutans (Mean±SD)×10^x^copy/mL	Lactobacillus (Mean±SD)×10^x^copy/mL
Mother First	(1,22±3,78)×10^6^	(0,34±0,74)×10^3^
Mother Second	(0,59±1,86)×10^6^	(1,29±6,34)×10^3^
P	0,328	0,353
Newborn First	(0,40±1,31)×10^3^	(0,10±0,60)×10^2^
Newborn Second	(2,81±6,04)×10^3^	(0,92±2,33)×10^2^
P	0,013	0,043

**Table-V:** Distribution of infant’s mean S. mutans and LB counts based on baby-related factors investigated during the second sampling.

Variables at the 2^nd^ Sampling	Groups	N	Baby SM 2nd	Baby LB 2nd

			(Mean±SD)×10^3^copy/mL	(Mean±SD)×10^2^copy/mL
Erupted Tooth	Yes	16	(5,98±8,12)×10^3^	(0,55±1,60)×10^2^
	No	24	(0,69±2,66)×10^3^	(1,16±2,71)×10^2^
	P		0,005	0,425
Primary Care Giver	Mother	34	(3,21±6,46)×10^3^	(0,74±2,11)×10^2^
	Other	6	(0,56±1,38)×10^3^	(1,89±3,38)×10^2^
	P		0,329	0,271
Oral Hygiene Practices	Everyday	7	(0,89±1,36)×10^3^	(0,43±1,14)×10^2^
	≤ 3 times a week	17	(3,41±4,48)×10^3^	(0,61±2,05)×10^2^
	None	16	(3,01±8,39)×10^3^	(1,45±2,92)×10^2^
	P		0,651	0,495
Bathing	Twice a week	11	(2,39±3,80)×10^3^	(1,38±3,19)×10^2^
	Once a week	25	(3,42±7,18)×10^3^	(0,52±1,37)×10^2^
	Once every 10 to 15 days	4	(0,16±0,32)×10^3^	(2,09±4,18)×10^2^
	P		0,595	0,348
Feeding	Breast Milk	17	(1,95±3,39)×10^3^	(1,07±2,65)×10^2^
	Breast milk+ Complementary Food	23	(3,44±7,43)×10^3^	(0,80±2,11)×10^2^
	P		0,447	0,722
Shared Utensil Usage/ Kissing on the Lips	Yes	15	(2,67±4,65)×10^3^	(0,69±2,18)×10^2^
	No	25	(2,89±6,83)×10^3^	(1,05±2,44)×10^2^
	P		0,914	0,645
Bottle Usage	Yes	17	(4,65±8,61)×10^3^	(1,08±2,41)×10^2^
	No	23	(1,45±2,48)×10^3^	(0,79±2,31)×10^2^
	P		0,098	0,699
Pacifier Usage	Yes	11	(3,73±4,91)×10^3^	0±0
	No	29	(2,46±6,46)×10^3^	(1,26±2,66)×10^2^
	P		0,558	0,126
Sugary Food Consumption	Yes	28	(3,35±7,02)×10^3^	(1,12±2,69)×10^2^
	No	12	(1,54±2,39)×10^3^	(0,43±1,03)×10^2^
	P		0,391	0,397

SM: S. mutans; LB; Lactobacillus spp.

## DISCUSSION

In the present study, we investigated the presence of *S.mutans* and *LB* in mothers and newborns using the qRT-PCR method and correlated the findings with various maternal- and infant-related factors. In the past researchers have suggested that the colonization of *S.mutans* in children occurs during the period known as the “window of infectivity” and cannot occur before the eruption of teeth.^10^-^12^ However, we observed the presence of *S.mutans* DNA in the saliva samples of 37% of the edentulous newborns and 29% of the edentulous six-month-old infants. Prior studies conducted with different microbiological methods also found that *S.mutans* can be detected in edentulous infants.^4^,^13^-^16^ While Tanner et al.^14^ suggested that of *S.mutans* can colonize on the tongue’s surface, Wan et al.^13^ suggested that presence of *S.mutans* could be associated with a Bohn nodule.

In their studies, both Carlsson et al.^17^ and Tanner et al.^14^ suggested that the presence of *LB* in children’s mouth is associated with carious lesions. In contrast with these studies, we found *LB* DNA in 5% of the newborns’ saliva samples and 17% of the six-month-old edentulous infants. Similarly, Plonka et al.^16^ and Hegde et al.^18^ found *LB* DNA in the edentulous newborns and Hegde et al.^18^ attributed this to maternal vaginal flora. However, they could not associate these findings with delivery type.

It has been suggested that the bacteria responsible for dental caries, along with endogenous bacteria, often show a transition from the primary carer, who is generally the mother, to the baby, which is referred to as vertical transmission.^19^,^20^ In the present study, we were unable to correlate the increases in the *S.mutans* and *LB* counts with any of the maternal factors. However, we did identify a statistically significant relationship between the presence of erupted teeth and an increase in the *S.mutans* count. Rosenblatt et al.^21^ reported no association between newborns’ salivary microflora and ‘maternal and infantile’ factors such as the mother’s sociodemographic status and feeding attitudes, baby’s gender, and baby’s birth weight. Similarly, Ruiz-Rodriguez et al.^22^ also could not identify a direct link between maternal levels of *S.mutans* colonization in relation to infants’ *S.mutans* counts. Nonetheless, some studies have reported contrary results, indicating that maternal factors can influence infants’ S. mutans colonization.^5^,^16^,^23^ One such study, by Plonka et al.^16^ associated the presence of S. mutans in the neonatal period with maternal S. mutans counts of >10^5 CFU/ml.

In the present study, the delivery type was not found to have a significant effect on either *S.mutans* or *LB* colonization. Ubeja et al.^24^ and Thakur et al.^25^ similar to our study, argued that the delivery type does not affect *S.mutans* colonization, whereas Li et al.^26^ Suggested that the delivery type could influence the oral microflora. Wassel et al.^4^ Found that infants delivered via C-section showed higher acquisition of *S.mutans* when compared with infants delivered via vaginal delivery. By contrast, Pattanaporn et al.^27^ Reported that vaginally delivered children exhibited higher acquisition of *S.mutans*. This discrepancy may be attributed to differences in the age distribution of the sample groups in the studies as well as to variations in the methods chosen for the *S.mutans* quantification. In this study, the infants’ feeding method also did not have a statistically significant effect on the *S.mutans* and LB counts. However, Wassel et al.^4^ and Thakur et al.^25^ found infants fed with bottles to have significantly higher *S.mutans* colonization, whereas Li et al.^5^ did not find a significant relationship in their study.

### Limitations:

It include the restricted diversity of sociodemographic variables, as the study was conducted at a single university. Additionally, to better evaluate the relationship between the oral flora of newborns and their mothers with sociodemographic characteristics, it may be necessary to follow them in later stages of their lives.

## CONCLUSION

Our results indicated that *S.mutans* and *LB* were able to colonize the oral mucosal surfaces of edentulous infants. The increased counts of both bacteria of infants could only be related with tooth eruption but the importance of mothers in relation to the development of infants’ oral flora cannot be overlooked.

## Data Availability Statement:

None.

### Author Contributions:

**HK** and **CA:** Conceptualization, data curation, formal analysis, investigation, methodology, validation.

**HK** and **AC:** Validation, writing-original draft, and writing - review and editing.

All authors gave their final approval on the version that will be published, agreed to be accountable for all aspects of the work in ensuring that questions related to the accuracy or integrity of any part of the work are appropriately investigated and resolved.
